# Medical News

**Published:** 1856-11

**Authors:** 


### THE MEDICAL EXAMINER.

PHILADELPHIA, NOVEMBER, 1856.

### MEDICAL NEWS.

We regret to be obliged to state that Professor Mütter has left the country without fulfilling his generous intentions toward the College of Physicians of Philadelphia, mentioned by us in a previous number.

We have received the first four numbers of "The Medical World," a weekly journal, published in Boston, and edited by Dr. J. V. C. Smith. As the whole journal is strongly redolent of quackery, in its advertisements, articles and editorials, we decline exchanging with it.

We are glad to see that Professor George C. Blackman, of the Medical College of Ohio, has become one of the editors of the Western Lancet.

Dr. Montgomery has resigned the Professorship of Midwifery in the School of Physic in Ireland, which he has for the long space of twenty-nine years so ably filled.

A Supplement to the Pharmacopœia of 1850 has been issued by the College of Physicians in Ireland, that all medicines intended for external use should be dispensed in angular vessels, and that the more active and poisonous preparations and articles of the Materia Medica, shall be kept and sold in similar bottles or vessels, while those intended for internal use shall be dispensed in round bottles or vessels, and also the less active simples and compounds kept and sold in round vessels, the intention being, that the touch alone may be sufficient to distinguish vessels containing articles possessed of dangerous qualities.—*Med. Times and Gaz.*

Medical periodicals in Europe.—A computation, the accuracy of which may be relied upon, gives the following statistics of the number of journals of medicine and pharmacy now published in the different languages of Europe :—German language, 58; Dutch, 8; Swedish, 8; English, 30; French, 47; Italian, 12; Spanish, 9. These languages do not correspond each to a distinct nationality; thus, the Belgian journals are included in the same category as the French, and amount to the not inconsiderable number of 13.—*Arch. Gen. de Medecine.*

---

Irish Analysis of the Adulterations of Whiskey.—An Irish analyst, in the *Limerick Chronicle*, gives the following composition of adulterated whiskey:—"It is composed," he says, "of one-third spirits, one-third aqua-fortis, one-third vitriol, and one-third water." The writer in the *Times*, who quotes the paragraph, suggests that the analyst who penned the statement, may probably have left one half of his senses in his nightcap, lost one half in taking the dram which he analyzed, and made use of the rest in obtaining his results.

---

The resignation of his Chair of Physiology by Dr. Carpenter, which was doubted by the *Medical Times*, is now stated on authority by the *Lancet.* Dr. Hughes Bennet of Edinburgh recently visited the Museum in connection with the chair held by Dr. Carpenter, which gave rise to some surmises of Dr. Bennet (so unwisely rejected in Edinburgh) coming to London; but we hear it said there is no foundation for the rumor. A severe loss to physiological science is experienced in London by Dr. Carpenter giving up physiology.—*Dublin Med. Press.*

---

Extensive Injuries during Pregnancy.—In this city last, winter, a robust German female, about 26 years of age, and five months pregnant, fell into a well, and descended 51 feet! She suffered an oblique fracture of the thigh, complete dislocation at the knee-joint, and a fracture of the tibia and fibula just above the ankle! At no time after the accident did she manifest any signs of abortion, but went her full time, and was delivered, some time in June last, of a well-formed, healthy child. It may not prove uninteresting to mention that, during the pregnancy, the fracture in the vicinity of the ankle-joint failed to unite. After delivery, the process of reparation commenced, although slowly, and she is now regaining the use of her limb.—*Dr. H. Tyler Smith's Obstetric Lectures in Lon. Lancet.*

*Abstract of Meteorological Observations for September, 1856, made in the Twenty-fourth Ward, Philadelphia.* By PROF. JAMES A. KIRKPATRICK.

| 1856. September. | BAROMETER. Reduced to 32°. Daily Mean | Mean Daily Range. | THERMOMETER. Daily Mean | Mean Daily Range | Extreme Range | Solar Radiation, 2 P.M | Rel. Humid. 2 P.M. | Force of Vapor 2 P.M. | Dew Point 2 P.M. | Rain | Prevailing Winds. | Remarks. |
|---|---|---|---|---|---|---|---|---|---|---|---|---|
| | Inches. | Inches. | Deg. | Deg. | Deg. | Deg. | Hunds. | Inches. | Deg. | Inches. | Points. | |
| 1 | 29.806 | .038 | 59.0 | 7.3 | 13 | | 84 | .577 | 63.1 | | NE. | Morning and afternoon cloudy; evening clear. |
| 2 | 29.817 | .016 | 62.3 | 3.3 | 26 | 23 | 37 | .335 | 48.0 | | NW. | Clear. |
| 3 | 29.866 | .050 | 63.0 | 1.3 | 26 | 25 | 45 | .401 | 52.9 | | SW. | Morning and afternoon cloudy; evening clear. |
| 4 | 29.906 | .040 | 69.0 | 6.0 | 30 | 26 | 46 | ·442 | 55.5 | | (Var.) | Clear. |
| 5 | 29.934 | .028 | 67.0 | 3.3 | 27 | 39 | 47 | .487 | 58.3 | | S. | M. and ev. clear; aft. cloudy. *Barometer highest* 29.957. |
| 6 | 29.857 | .077 | 71.5 | 7.2 | 20 | 13 | 64 | .579 | 63.2 | 0.004 | SSW. | Cloudy; evening *rain*. |
| ☽ 7 | 29.767 | .090 | 75.7 | 4.2 | 21 | 43 | 57 | .689 | 68.2 | | SW. | Morning and afternoon cloudy; evening clear. |
| 8 | 29.739 | .035 | 74.7 | 1.0 | 21 | | 68 | .788 | 72.2 | 0.231 | SW. | Cloudy; morning and afternoon *rain*. |
| 9 | 29.887 | .148 | 73.3 | 2.7 | 21 | 44 | 42 | .460 | 56.7 | | (Var.) | Clear. |
| 10 | 29.709 | .178 | 73.0 | 1.0 | 26 | | 60 | .677 | 67.7 | | SE. | Morning heavy *fog*; m. and aft. clear, ev. cloudy. |
| 11 | 29.535 | .174 | 79.2 | 6.2 | 20 | 26 | 47 | .667 | 67.2 | 0.314 | SW. | Cloudy; afternoon *rain, thunder and lightning*. *Thermometer highest* 90°. |
| 12 | 29.652 | .117 | 69.0 | 10.2 | 20 | 7 | 49 | .456 | 56.4 | | (Var.) | Cloudy. |
| 13 | 29.552 | .100 | 70.3 | 1.3 | 23 | 10 | 61 | .587 | 63.6 | | W. | Morning cloudy; afternoon and evening clear. |
| ○ 14 | 29.688 | .137 | 65.7 | 4.7 | 22 | 21 | 43 | .362 | 50.1 | | N. | Clear. |
| 15 | 29.672 | .022 | 68.7 | 3.0 | 25 | 15 | 56 | .483 | 58.0 | | SE. | Morning and evening cloudy; aft. clear. |
| 16 | 29.704 | .036 | 65.0 | 3.7 | 18 | 28 | 36 | .299 | 45.0 | | NNW. | Clear. |
| 17 | 29.728 | .066 | 68.7 | 9.7 | 30 | | 51 | .523 | 60.3 | | NW. | Clear. |
| 18 | 29.607 | .121 | 79.3 | 10.7 | 27 | 33 | 50 | .677 | 67.7 | | (Var.) | Morning cloudy; afternoon and evening clear. |
| 19 | 29.692 | .085 | 67.7 | 11.7 | 18 | 39 | 42 | .388 | 52.0 | | SW. | Cloudy. |
| 20 | 29.677 | .019 | 63.3 | 5.7 | 8 | | 89 | .591 | 63.7 | 1.538 | SW. | Cloudy, morning *rain, thunder* and *lightning*. |
| ☾ 21 | 29.637 | .046 | 66.0 | 3.3 | 18 | 16 | 67 | .545 | 61.4 | | NE. | Cloudy. |
| 22 | 29.547 | .090 | 58.0 | 8.0 | 7 | | 82 | .426 | 54.6 | 0.018 | NNW. | Morning and aft. cloudy; evening clear; aft. *rain*. |
| 23 | 29.478 | .069 | 56.0 | 4.7 | 16 | 16 | 29 | .175 | 31.2 | 0.016 | NW. | Morning and evening cloudy; afternoon clear; night *rain*. |
| 24 | 29.543 | .089 | 51.8 | 4.2 | 13 | 22 | 42 | .202 | 34.7 | | W. | Morning cloudy; afternoon and evening clear. |
| 25 | 29.671 | .128 | 53.0 | 5.8 | 25 | | 48 | .284 | 43.6 | | SSW. | *Hoar frost*, first observed. Clear. *Thermometer lowest* 39°. |
| 26 | 29.630 | .041 | 59.7 | 6.3 | 29 | 25 | 45 | .339 | 48.3 | | S. | Clear. |
| 27 | 29.663 | .034 | 61.7 | 2 0 | 22 | 30 | 38 | .313 | 46.2 | | NW. | Clear. |
| ● 28 | 29.697 | .035 | 64.3 | 2.7 | 22 | | 59 | .475 | 57.6 | | (Var.) | Morning and evening clear, afternoon cloudy. |
| 29 | 29.549 | .098 | 66.0 | 1.7 | 19 | | 63 | .510 | 59.6 | | NE. | Morning and afternoon cloudy, evening clear. |
| 30 | 29.334 | .265 | 63.0 | 7.0 | 14 | | 95 | .650 | 66.5 | 3.531 | SW. | Cloudy; m. and aft. *heavy rain*. *Bar. lowest* 29.313. |
| Means for Sept. 1856 | 29.686 | .082 | 66.2 | 5.0 | 20.9 | 25.1 | 55 | .480 | 56.4 | 5.702 | S. 73° 30′ W. 189. | |

